# Neurocentrist identity theory and neuro-phenomenal typing: A commentary on Manzotti's, “The boundaries and location of consciousness as identity theories deem fit”

**DOI:** 10.3389/fpsyg.2022.1058325

**Published:** 2022-11-15

**Authors:** Tomáš Marvan, Michal Polák

**Affiliations:** ^1^Department of Analytic Philosophy, Institute of Philosophy, Czech Academy of Sciences, Prague, Czechia; ^2^Department of Philosophy, University of West Bohemia, Pilsen, Czechia

**Keywords:** identity theory, phenomenal types, neural types, neural correlates of consciousness, quality spaces

## Introduction

Manzotti (2021) surveys recent variants of identity theories, defending his own preferred version, mind-object identity theory (MOI). According to this view, experiences are identical with the external objects, and the mind is thus literally “spread” in the world. Manzotti supports this view with considerations about indiscernibility of properties and other theoretical considerations. He claims that brain-mind accounts of identity commit the “fallacy of the center,” locating conscious mind inside the skull. Amongst other recent works, he comments on our (Polák and Marvan, 2018) article, in which we defended a standard, neurocentrist version of type identity theory, and supplemented it with a sketch of neuro-phenomenal typing. Manzotti holds that although we appeal to neuro-phenomenal types in our account of mind-brain identity, we nevertheless “lack a convincing explanation as to why the type of neural processes should be identical to the type of one's experience.” This is a fair point. We didn't do much in our 2018 article to support our views on the principles of neuro-phenomenal typing, either by detailed theoretical considerations or by empirical evidence. In this short rejoinder, we offer the missing argument. By doing so, we also respond both to Manzotti's cited objection and to the charge of the “fallacy of the center.”

The idea of neuro-phenomenal typing is not just haphazard, or justified only by common-sense intuitions (as it mainly was in our 2018 article), but can be directly supported by empirical observations. To show this, we draw on Pautz (2017) who argues that similarity and difference relationships between sensory experiences do not match similarity and difference relations between the physical structures of perceived objects. Pautz uses this argument to criticize naïve realism in the philosophy of perception. Naïve realism shares Manzotti's view that experienced qualities belong to external objects, not to the states of the central nervous system. And although such a view is controversial, it would seem reasonable to expect that similarities and differences between experiences track the similarities and differences between worldly objects. However, the research to which Pautz draws attention challenges this expectation. In his words:

Psychophysics has shown that, even in normal cases, qualitative similarity is very poorly correlated with external physical similarity. At the same time, neuroscience has shown that neural similarity is the only accurate predictor of qualitative similarity. In short, the typical situation is that there is “good internal correlation” even while there is “bad external correlation” (Pautz, 2017, p. 25).

By “normal cases,” Pautz means cases in which the subjects do not hallucinate or suffer from perceptual illusions.

Manzotti asks for an explanation of why types of neural processes are identical to types of experiences. The reason is precisely the “good internal correlation” Pautz talks about. A commitment to a tight neuro-phenomenal coupling, such as the one to be found in our 2018 article, has a better chance than MOI at getting the empirical facts right. The close correspondence between neural and phenomenal types, in itself, cannot prove that identity theory is correct. But Manzotti does accept the correctness of the identity theory, and disputes only the precise location of the mind. We therefore do not attempt to defend the identity theory here. Rather, we claim only that identity theorists should continue to locate the mind in the skull, because that is where, according to evidence we review, it resides.

## Evidence for tight neuro-phenomenal pairing

Pautz (2017) briefly surveys examples from visual, olfactory and auditory neuroscience. The findings he refers to, which we supplement by references to other recent research, are remarkably coherent. They all indicate that similarities and differences among experiences are grounded in patterns of neural responses rather than in the physical make-up of objects eliciting the experiences. This is not good news for views such as Manzotti's which build the metaphysical identity relation on the correspondences between physical constitution of things and experiences.

Pautz notes that reflectance profiles of blue surfaces closely match the reflectance profiles of green surfaces and do not match well the reflectance profiles of purple surfaces. Experiences of blue, though, are felt as more similar to purple, not green. The conscious character of color experiences is thus not determined by external physical properties, such as reflectance profiles, as MOI and naïve realism assume. Rather, the character of color experiences corresponds to the patterns of neural responses to visual stimuli. In the case at hand, the patterns are located in area V4 of the visual cortex and in other sites that contribute to color processing.

Turning to olfaction, conscious smells can display similarities even when prompted by odorants with very different molecular structure. Sell (2006) gives the example of musks, a family of experientially homogeneous class of odorants which widely differ in their physical structure. Similarly, some odorants clustered into “functional groups,” such as esters, produce distinctly similar smells despite variance in their molecular composition. Again, such disparities disappear when the researchers start comparing the olfactory experiences with patterns of neural responses to odorants. Pautz refers to the work of Howard et al. (2009) who show that spatially distributed ensemble activations in human posterior piriform cortex coincide with perceptual ratings of odor quality. Guerrieri et al. (2005) reached a similar conclusion. They measured honeybee olfactory responses to 16 different odorants, while simultaneously performing optophysiological recordings of activations in antennal lobe, an insect olfactory center comparable in its function with the olfactory bulb of the vertebrates. They found that physiological distances uncovered by these recordings correlate well with the perceptual distances in the honeybee olfactory quality space, and can be reliably used to predict such distances (and vice versa).

Auditory research provides additional examples of the same phenomenon. As Pautz (2017, p. 27) points out, experienced properties of sounds correlate only in complicated, non-linear and multivariable ways with the physical constitution of auditory stimuli. On the other hand, they neatly correlate with the overall magnitude of neural response in cortical auditory centers. Going beyond the classical sensory modalities that Pautz discusses, research on pain perception further corroborates the neuro-phenomenal hypothesis. Coghill et al. (2003) investigated interindividual differences in pain perception between controls and highly sensitive individuals. They found that the only difference-making factor between these two groups lies in the cortex. The physical characteristics of stimuli are strictly matched between the subjects, and there are no significant interindividual differences in pain processing prior to the cortex. Only primary somatosensory cortex, anterior cingulate cortex and prefrontal cortex were activated more frequently and robustly in the highly sensitive individuals. Identifying the pain experience with anything else but the cortical activations thus precludes explaining the difference between controls and highly sensitive individuals. Moreover, it is not even clear what physical object, in Manzotti's sense, is to be identified with pain experience.

## Discussion

Manzotti's MOI must be committed to sorting experiences into types by physical features of objects because he claims that these physical features are identical to experiences. If the physical features of objects differ, then surely the experiences identical with them must differ as well. But if experiences are typed by the physical features of the external objects and independently of neural responses to these objects, what accounts for the convergent empirical results summarized in the previous section? Why the differences between types of experiences in multiple sensory domains seem to track the properties of neural responses to stimuli, and not the physical constitution of the stimuli? If we bracket the brain's contribution to sorting experiences into types, it would seem that we are left with no serious explanation of similarities and differences between experiences. In contrast, if we accept Pautz's argument about close neuro-phenomenal correspondences, we obtain “a natural explanation of the similarity relations between the conscious experiences” (Papineau, 2021, p. 21).

A recent wave of “neuro-phenomenal structuralism” (Fink et al., 2021; Lyre, 2022; see also Malach, 2021, for related approach) explores the systematic correspondences between types of experiences and types of neural responses in yet another fruitful way. Neuro-phenomenal structuralists argue that the study of neural correlates of consciousness (NCCs) must go beyond finding mere co-activations of localized brain structures and experiences. Instead, they propose, we need to acknowledge a “structural constraint” on NCCs, so that mere statistical correlations between experiences and brain processes are ruled out and proper NCCs are identified. Neural activation satisfies the structural similarity constraint if it preserves the structure of similarities and differences governing the domain of experiences it is associated with. In other words, the similarity and difference relations between neural activations must mirror the similarities and differences between experiences corresponding to these activations. Neuro-phenomenal structuralists draw on the work of Brouwer and Heeger (2009), to which Pautz (2017) also appeals in his discussion of color experiences. We believe that examples from other sensory domains discussed above provide further support for the neuro-phenomenal research programme.

To forestall a possible misunderstanding of our position, we do not wish to claim that systematic differences in the physical structure of external objects have no causal bearing on the differences between experiences. Rather, the claim is that the experiential differences are ultimately fixed by the properties of patterned neural responses to the external physical differences, not by these external differences themselves. Modern research on olfaction provides an illustration of this phenomenon. As Barwich (2020, esp. chaps. 3–7) shows, finding a tight match between the molecular structure of odorants and the structure of olfactory experiences is a daunting task. A shift from the molecular structure itself to the evoked activation patterns in the central nervous system paints a clearer picture. The example of Guerrieri et al. (2005), mentioned above, is instructive. The researchers found an overall match between aspects of physical structure of odorants and olfactory experiences. In particular, they identified the two main orthogonal dimensions of the bee olfactory space: functional group of the odorants (such as primary and secondary alcohols, aldehydes or ketones) and their carbon length (from six to nine carbons). However, when they account for experiential similarities driving similar discriminative reactions in the honeybees, they directly appeal to similar patterns of glomerual activations. For instance, a strong behavioral generalization between the functional groups of primary and secondary alcohols can be readily explained by “extremely similar” activation patterns in the antennal lobe in the studied honeybees (Guerrieri et al., 2005, p. 727).

All identity theories, not just Manzotti's MOI but also the one we favor, contain unpalatable elements. The greatest challenge for our preferred version of identity theory is to explain how properties of experiences can literally be the same as the properties of brain states. While this short rejoinder does not decisively answer that challenge, we at least show that identifying experience not with brain states but with the physical objects themselves, as Manzotti suggests, runs counter to increasing amount of empirical evidence. Beyond the evidence for close neuro-phenomenal correspondences, around which we built this response, one could also cite other factors that highlight the role of physical stimulus-independent neural processes shaping the resulting conscious percepts. For example, Vetter and Newen (2014) suggest that non-perceptual top-down mechanisms may enter into visual processing and alter or otherwise influence the resulting visual experience independently of physical features of external objects.

In conclusion, the close type-type correspondences between experiences and brain states indicate that identifying experiences with their associated neural activations is not a fallacy, as Manzotti claims, but a metaphysically and methodologically sound strategy.

## Author contributions

All authors listed have made a substantial, direct, and intellectual contribution to the work and approved it for publication.

## Funding

Authors were supported by the Czech Science Agency (GACR), project no. 20-14445S (Dual Models of Phenomenal Consciousness).

## Conflict of interest

The authors declare that the research was conducted in the absence of any commercial or financial relationships that could be construed as a potential conflict of interest.

## Publisher's note

All claims expressed in this article are solely those of the authors and do not necessarily represent those of their affiliated organizations, or those of the publisher, the editors and the reviewers. Any product that may be evaluated in this article, or claim that may be made by its manufacturer, is not guaranteed or endorsed by the publisher.
